# Altered levels of transthyretin in human cerebral microdialysate after subarachnoid haemorrhage using proteomics; a descriptive pilot study

**DOI:** 10.1186/s12953-023-00210-z

**Published:** 2023-07-07

**Authors:** Fredrik Ginstman, Bijar Ghafouri, Peter Zsigmond

**Affiliations:** 1 Department of Neurosurgery in Linköping and Department of Biomedical and Clinical Sciences, Linköping University, Linköping, Sweden; 2 Pain and Rehabilitation Center and Department of Health, Medicine and Caring Sciences, Linköping University, Linköping, Sweden

**Keywords:** Proteomics; subarachnoid hemorrhage; microdialysis; transthyretin; prealbumin; biomarkers; brain ischemia

## Abstract

**Background:**

Subarachnoid haemorrhage (SAH) is one of the most severe forms of stroke in which delayed cerebral ischemia is one of the major complications. Neurointensive care aims at preventing and treating such complications and identification of biomarkers of early signs of ischemia might therefore be helpful.

**Methods:**

We aimed at describing proteome profile in cerebral microdialysate in four patients with aneurysmal SAH using two dimensional gel electrophoresis in combination with mass spectrometry in search for new biomarkers for delayed cerebral ischemia and to investigate if there were temporal fluctuations in those biomarkers over time after aneurysmal bleed.

**Results:**

The results showed transthyretin in nine different proteoforms (1001, 1102, 2101, 3101, 4101, 4102, 5001, 5101, 6101) in cerebral microdialysate samples from four patients having sustained SAH. Several proteoforms show highly differing levels and pooled analysis of all samples showed varying optical density related to time from aneurysmal bleed, indicating a temporal evolution.

**Conclusions:**

Transthyretin proteoforms have not earlier been shown in cerebral microdialysate after SAH and we describe differing levels based on proteoform as well as time from subarachnoid bleed. Transthyretin is well known to be synthetized in choroid plexus, whilst intraparenchymal synthesis remains controversial. The results need to be confirmed in larger studies in order to further describe transthyretin.

**Supplementary Information:**

The online version contains supplementary material available at 10.1186/s12953-023-00210-z.

## Background

Subarachnoid haemorrhage (SAH) from aneurysm rupture comprises approximately 5% of all strokes and is considered to be one of the most severe forms of stroke with an incidence of 6/100 000 and overall mortality ranging from 32–67% [1]. Patients with aneurysmal SAH have well known risk for development of vasospasm which refers to a morphologic narrowing of the cerebral vessels and it may appear in 30–40% of patients. Cerebral vasospasm, among other pathophysiological mechanisms may lead to delayed cerebral ischemia (DCI) in 20–30% which might lead to severe disability [2, 3]. In fact, half of the patients surviving aneurysmal SAH suffer from long-term disability and about one third remain dependent [1, 4].

Neurointensive care aims at detecting early signs of DCI in order to prevent and treat underlying mechanisms to this condition. Neurointensive care uses different monitoring techniques such as bedside neurologic examination, transcranial Doppler (TCD), Xenon-CT cerebral blood flow measurements, computerized tomography perfusion (CTP) and microdialysis in order to detect early signs of ischemia, the summary of techniques often conceptualized as multimodality monitoring [5–8]. Brain microdialysis enables in vivo sampling of interstitial metabolites and compounds by means of thin intraparenchymal catheters inserted to the brain [9].

Using brain microdialysis, well-known metabolites indicating metabolic stress or ischemia are lactate/pyruvate-ratio and glucose [10]. Some evidence also exists that lactate together with excitatory amino acids and a few other proteins may indicate worse prognosis after aneurysmal SAH [11]. Although a number of other metabolites have been described as biomarkers indicating pending ischemia, the majority have been detected in blood and CSF [12]. Studies on biomarkers in cerebral microdialysate, analyzed by proteomic technique, are scarce [13].

There is however a need for further markers for ischemia and metabolic stress to indicate and at best prognosticate which patients are at risk of developing DCI. Ideally, biomarkers could indicate pending ischemia at an early stage, before irreversible neuronal damage has appeared. Previous studies on cerebral microdialysate, analyzed by proteomic technique by Maurer et al. have for example shown an increase in protein concentrations of several proteoforms of glyceraldehyde-3-phosphate dehydrogenase (GAPDH) in patients who later developed symptomatic vasospasm, whereas heat–shock cognate 71 kDa protein (HSP7C) proteoforms were decreased [14].

In this pilot study we aimed at describing proteome profile in cerebral microdialysate in patients with aneurysmal SAH using two dimensional gel electrophoresis followed by identification by liquid chromatography tandem mass spectrometry (LC–MS/MS) in search for new biomarkers for delayed cerebral ischemia and to investigate if there are temporal fluctuations over time after aneurysmal bleed. A secondary aim was to try to confirm the findings of Maurer et al.

## Methods

### Ethics

The regional ethics committee in Linköping. Sweden as well as Swedish Ethical Review Authority approved the study protocol (decision number 2013/471–31 and 2019–06444). The study was carried out in accordance with relevant guidelines and regulations, including the WMA Declaration of Helsinki.

### Patients

We describe a group of four patients, two men and two women, with SAH treated with surgical clipping (3 patients) and endovascular coiling (1 patient). Mean age were 56 years. All patients were monitored with standard neurointensive care methods including microdialysis monitoring which is routine monitoring at the Dept. of Neurosurgery in Linköping. In all four patients, apart from bedside analysis of routine metabolites, we undertook proteomic analysis of the microdialysis samples as described below. DCI was defined as new neurologic deficits that could not be explained by other reasons. As the samples were from patients from routine microdialysis monitoring at the department, the inclusion criteria were; 1) microdialysate samples should be available from all time points, 2) the same perfusion fluid should have been used in all patients and 3) the samples should have been collected and stored during the same time frame. All those criteria are important factors that affect protein recovery in microdialysis samples when performing proteomic analysis.

### Patient characteristics

#### Patient 1

Fifty-six year old male, otherwise healthy. Presents unconscious with right sided intracerebral hematoma and SAH with intraventricular engagement. Glasgow coma scale (GCS) 5 on admission. He was operated on the same day with clipping of pericallosal aneurysm and evacuation of hematoma. No clinical or radiological signs of DCI or vasospasm during inpatient care.

#### Patient 2

Sixty-eight year old male. History of hypertension and asthma. Earlier the same year presented with SAH from anterior communicant aneurysm, treated with endovascular coiling and secondary DCI and vasospasm. Four months later patient presents with re-bleeding from the same aneurysm and focal intracerebral hematoma. GCS 6 admission. Operated on the same day with clipping of anterior communicating artery aneurysm. No clinical or radiological signs of DCI or vasospasm during the latter episode.

#### Patient 3

Fifty-one year old female. Earlier history of asthma and epilepsy, medicating with Lamotrigine. Presents unconscious, GCS 7 with SAH from anterior communicating artery aneurysm that is being operated on with microsurgical clipping the same day. Develops DCI with severe vasospasm from day 4 post ictus. Treated with intra-venous Nimodipine, induced hypertension and intra-arterial Nimodipine and balloon-angioplasty in a total of six times during her stay in the neurointensive care unit (NICU).

#### Patient 4

Fifty-one year old female, otherwise healthy. Presents unconscious, GCS 10 with SAH from anterior communicating aneurysm. Treated the day after by endovascular coiling of the ruptured aneurysm and another incidental aneurysm. No signs of DCI or vasospasm.

### Microdialysis

Microdialysis catheters with a membrane length of 10 mm and a molecular weight cut-off (MWCO) of 100 kDa (CMA-71, M-dialysis AB, Solna, Sweden) were used. The catheters were perfused with 3.5% human albumin in a water solution containing the excipients sodium chloride, N-acetyl-DL-tryptophan and caprylic acid (Albunorm. 50 g/l, Octapharma AB, Stockholm, Sweden), with addition of 30 mmol Urea (APL) at a rate of 0.3 µL/min using the CMA 106 perfusion pump (M-Dialysis AB, Solna, Sweden)[15, 16]. The first 2 h of sampling were discarded according to local routines and consensus praxis [10]. Samples were collected every 2 h for routine analysis of small molecular metabolites [17].

### Sample preparation

The dialysate samples were analyzed for the clinically routine analysis of metabolites immediately and then the samples were stored at -20 °C until transport to a -86 °C freezer at the laboratory. In order to investigate the feasibility of applying proteomic to investigate protein changes over time, we used a pool of samples from four patients, divided into 3 groups depending on timing of sampling after catheter insertion. The timeframes were set at early: Early phase mean 53 h after insertion, intermediate phase mean 59 h after insertion, late phase mean 76 h after insertion. Clinically it is known that the risk of developing vasospasm and DCI is highest between day 4–10 after SAH and we wanted to see whether these markers changed over time. Then other samples from the four included patients were analyzed individually according to the timeframe described above.

### Proteomic analysis

The perfusion fluid contained high amount of albumin therefore the samples were depleted using albumin & IgG Depletion column (GE Healthcare, Uppsala, Sweden) according to the manufacturer´s recommendation [17]. The depleted dialysate samples were desalted using 3 kDa Amicon spin column (Merck Millipore, Darmstadt, Germany) according to the manufacturer’s recommendation. The desalted samples were dried using speed vacuum concentrator and the dried proteins were dissolved in urea buffer solution (9 M Urea, 4% CHAPS, 65 mM DTT, 0.1% Bromophenol blue, 0. 2% Pharmalyte 3–10). The total protein concentrations in each sample were measured using 2-D quant kit (GE Healthcare, Little Chalfont, UK).

Fifty microgram protein was used for protein separation by 2-DE as described earlier [17]. Briefly the denatured proteins were applied to an immobiline dry strip polyacrylamide gel (IPG) pH 3–10. Proteins were separated according to the isoelectric point (pI) using Ettan IPGphor 3 IEF System (GE Healthcare) for 38 000 Vhs overnight. The IPG strips were either analyzed immediately or stored at -86 °C until analysis.

The second-dimension separation was performed using a pre-casted 8–12% gradient sodium dodecyl sulphate polyacrylamide gel electrophoresis (SDS-PAGE) from GE Healthcare using a horizontal 2-DE set up on a Multiphore (GE Healthcare). Proteins were separated according to the molecular weight at 20–40 mA for about 5 h. Immediately after electrophoresis the gels were placed in a fixation solution containing 50% methanol and 5% acetic acid in MilliQ water and incubated at room temperature with gentle shaking overnight. The separated proteins were stained with silver staining as described previously [17]. The gels were visualized and digitized using a charged coupled device camera (VersaDoc Imaging system 4000 MP; BioRad Laboratories) combined with the software PDQuest Advanced version 8.0.1 (Bio-Rad Laboratories). The amount of protein in a spot was assessed as background-corrected optical density, integrated over all pixels in the spot and expressed as optical density.

The protein spot of interest was excised from the gel, detained and the proteins were in-gel digested by trypsin (Promega Biotech AB, Nacka, Sweden). The tryptic peptides were extracted from the gel, dried and resolved in 0.1% formic acid for further identification by mass spectrometry. The tryptic peptides were analyzed using a nano liquid chromatography system (EASY-nLC, Thermo Scientific, Waltham, MA, United States) with a C18 column (100 mm × 75 µM, particle size 5 µM, Agilent technologies, Santa Clara, CA, USA) coupled to an LTQ Orbitrap Velos Pro MS (Thermo Scientific). The flow rate was set to 300 nL/min and the gradient buffer contained 0.1% formic acid in water (buffer A) and 0.1% formic acid in acetonitrile (buffer B). Buffer B was used in a linear gradient (0–100%) for 30 min to separate the peptides. Acquired raw files were analyzed with the software MaxQuant (version 1.5.8.3) against the human Swissprot/UniProt database (downloaded 2017), with the following parameters; enzyme digestion with trypsin, the maximum number of missed cleavages 2, minimum peptide length 6, parent ion mass tolerance 4.5 ppm. fragment ion mass tolerance 0.5 Da. Fixed modification was carbamidomethylation of cysteine, and variable modifications were oxidation of methionine and N-terminal acetylation. These identification criteria together with molecular weight and PI of the spot were applied to identify the protein with majority of the peptides.

## Results

### Sample overview

Microdialysate samples were collected every two hours. To obtain sufficient samples volume for proteomic analysis samples were pooled. A pool of samples from the patient’s catheters from early (53 h), intermediate (59 h) and late (76 h) time point after catheter insertion was analyzed first to investigate the possibility to apply proteomics for analyzing protein markers. The individual catheter samples from all four patients were then analyzed.

### Transthyretin

**Fig. 1:**
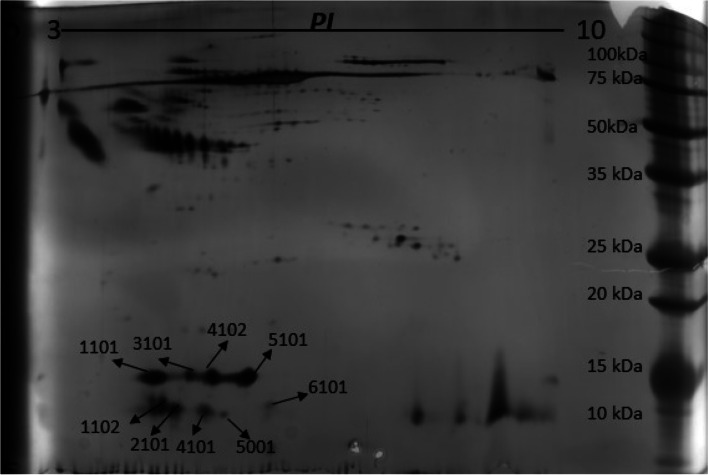
Representative 2-dimensional gel electrophoregram showing the pattern of different proteoforms of transthyretin. Arrows indicate spot numbers for different proteoforms of transthyretin presented in supplement Table S1

Our results show transthyretin in nine different proteoforms (1001, 1102, 2101, 3101, 4101, 4102, 5001, 5101, 6101) in cerebral microdialysate samples from four patients having sustained SAH. Different proteoforms were identified on gel by mass spectrometry and spot matching (uniprot.org/uniprot/P02766) (Fig. 1).

TTR-values for different proteoforms, see supplement Table S1.

**Fig. 2:**
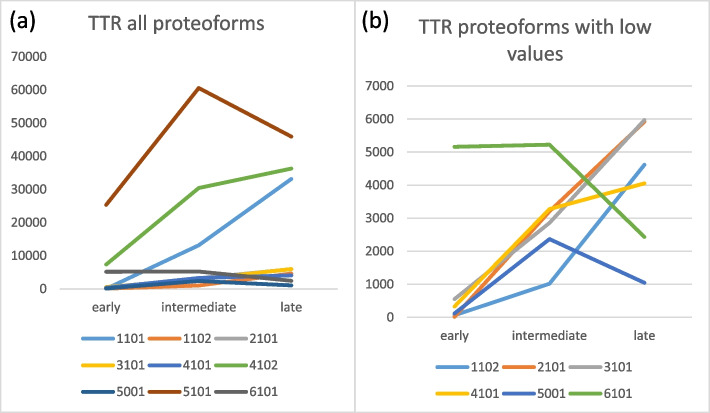
TTR-proteoforms optical density vary depending on time from aneurysmal bleed. X-axis refers to time from aneurysmal bleed and Y-axis refers to optical density. **a** TTR-proteoforms, all values **b** High resolution on TTR-proteoforms with low values

Some proteoforms (1101, 4102, 5101) show high optical density in many microdialysis samples while others (6101, 5001) show much lower or intermediate (1102, 2101, 3101, 4101) optical density. In a pooled analysis, different proteoforms seem to present different optical density related to time from aneurysmal bleed (Table 1 and Fig. 2a and b).

**Table 1:** Distribution pattern of different proteoforms related to time from aneurysmal bled (early, intermediate, late). Unit: Percentage optical density, dm = data missing

	1101	1102	2101	3101	4101	4102	5001	5101	6101
Early	18.2	62.7	14.5	552.1	326.8	7332.9	117.1	25,332.5	5160.7
Intermediate	13,139.1	1017.1	3206.4	2857.4	3276.1	30,412.8	2367.5	60,573.5	5225
Late	33,145	4618	5918.5	5972.1	4053.4	36,268.4	1045.1	45,926.9	2431.6

### Individual cases

Individual microdialysis catheters (no. 11) present low levels of some proteoforms (5001) and high levels of others (1102, 2101) while other catheters (no. 10) show almost as low levels of the first proteoform (5001) but apparently much lower levels of the latter (1102, 2101) (Table S1).

In one patient with severe vasospasm there is an obvious difference between the two catheter samples 8 and 9 from the left hemisphere and the single catheter sample from the right hemisphere 2 (Table 2). This patient developed a severe vasospasm, which according to the patient record was first diagnosed with decreased cerebral blood flow in the right hemisphere and later was verified as bilateral angiographic vasospasm. This finding indicates that there might be a difference transthyretin levels between different hemispheres related to vasospasm.

**Table 2:** Illustrating highly different optical density in many proteoforms between hemispheres hypothetically correlated to cerebral vasospasm. Unit: Percentage optical density, dm = data missing

	1101	1102	2101	3101	4101	4102	5001	5101	6101
8 left hemisphere	127,886.4	14,858.3	15,183	2126.3	dm	33,074.4	dm	70,509.8	dm
9 left hemisphere	6459.1	34,246.7	37,379	55,674.3	21,257	58,053.8	3453.5	15,482.1	214.3
2 right hemisphere	1947.2	1041.1	1643.7	1049.7	231.8	873.3	778.2	0	476.8

### GAPDH different proteoforms

**Fig. 3:**
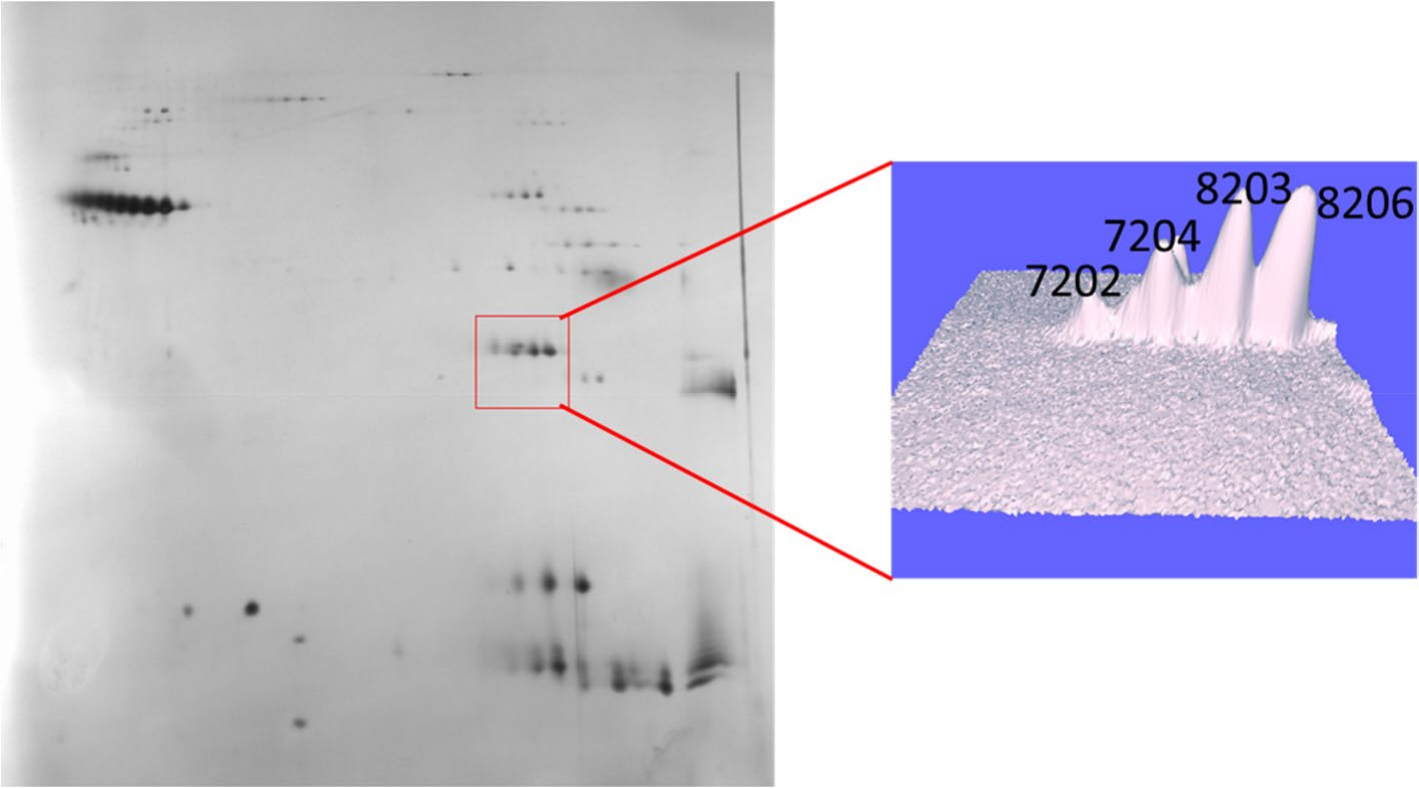
2-dimensional electrophoregram of microdialysis sample. The marked area represents proteoforms of GAPDH in a three-dimensional view

**Fig. 4:**
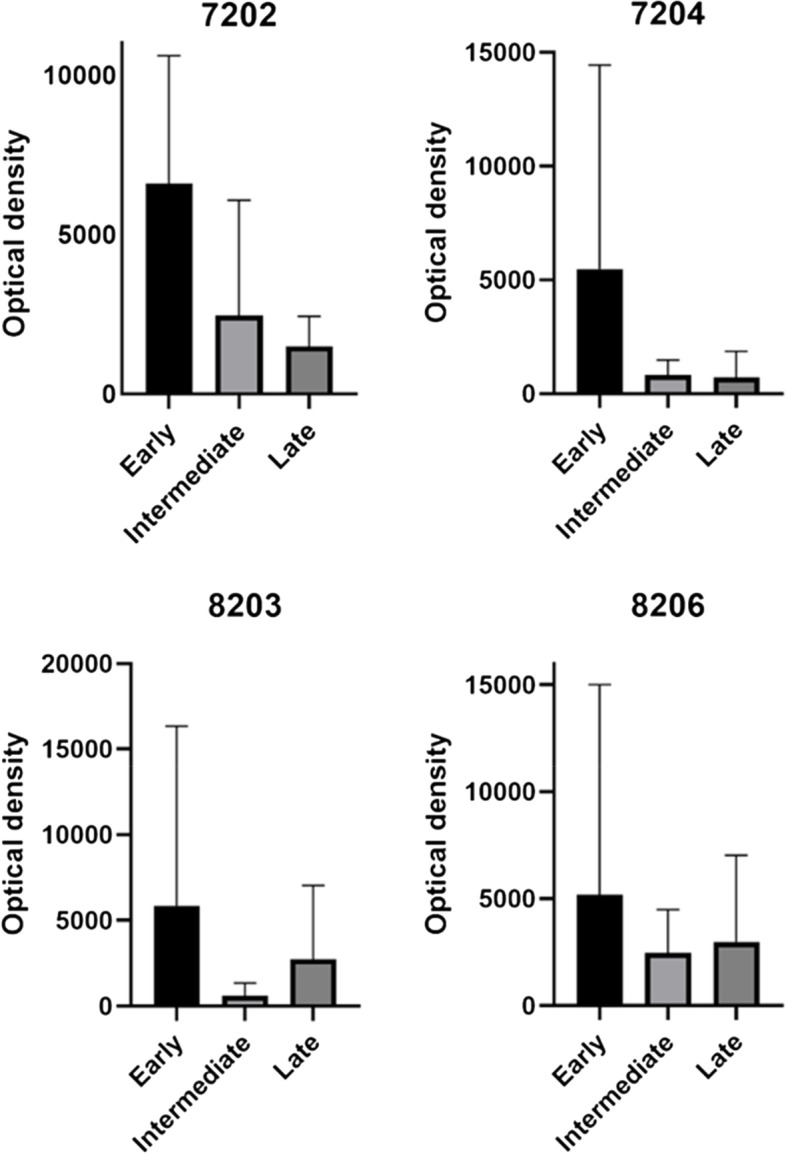
Illustrating mean values +—SD of optical density for GAPDH proteoforms in all patient catheters at three sampling times as earlier described

GAPDH was identified by spot matching based on previous results from Maurer et al. [14]. Four proteoforms were specified 7202, 7204, 8203 and 8206. In the pooled group analysis it appears that two of the proteoforms seem to decrease over time (7202 and 7204) while the other two seem to stay rather unchanged (8203 and 8206) (Figs. 3 and 4).

## Discussion

In this pilot study we aimed at describing the proteome profile in cerebral microdialysate in patients with aneurysmal SAH using two dimensional gel electrophoresis followed by identification by liquid chromatography tandem mass spectrometry (LC–MS/MS) in search for new biomarkers for delayed cerebral ischemia. Secondary aims were to investigate if any of these biomarkers show temporal fluctuations over time after aneurysmal bleed and to try to confirm the findings of Maurer et al. concerning GAPDH and HSP7C.

A number of studies have focused on using microdialysis as a monitoring tool after SAH but conflicting results exist whether standard metabolic parameters can predict delayed cerebral ischemia [18, 19]. However a recent review finds it promising while there is a need for multicenter studies focusing on interventions based on microdialysis findings [20].

In the search for new biomarkers Sarrafzadeh et al. showed increased levels of Matrix metalloproteinase-9 (MMP-9) in microdialysis samples during acute phase after SAH with significantly higher levels among patients that later developed DCI [21]. Maurer et al. has earlier investigated the proteome in a group of three ischemic stroke patients with cerebral microdialysis and showed presence of TTR among other proteins in the contralateral, non-injured, hemisphere [22]. TTR has also been described in microdialysis samples from muscle tissue in patients chronic myalgia [23].

Transthyretin (TTR) also named Prealbumin is a small 55 kDa globular protein involved in thyroxine and retinol binding and transport in serum and cerebrospinal fluid (CSF). It is synthesized by hepatic parenchymal cells and by the epithelial cells of choroid plexus of the brain and is the second most abundant protein in the cerebrospinal fluid [24]. Different proteoforms have been identified in CSF [25, 26].

It is generally considered as a negative acute phase reactant in serum, with low levels in acute inflammatory stress and trauma and low levels also correlated to negative nutritional status [27–29]. Serum TTR as nutritional marker seems to correlate to surgical method in SAH in a way that patients treated with surgical clipping shows increased energy expenditure and decreased TTR levels on day 4 after clipping. Increased catabolic state after surgery has been possibly associated with cerebral vasospasm [30, 31].

Serum-TTR has been suggested as a surrogate marker for blood–brain-barrier (BBB) disruption after TBI, however difficulties exist in relating to standard techniques for validation of BBB disruption [32, 33].

In brain, TTR it is related to development of Alzheimer’s disease or Amyloidosis but serum TTR has also been shown to correlate to prognosis and need for rehabilitation after ischemic stroke [34, 35]. TTR in CSF has been described as reduced in Guillain-Barré syndrome as well as in patients with meningioma [36, 37]. TTR has been shown to increase in cerebrospinal fluid after experimental brain ischemia in mice [38] and neuroprotective and neuroregenerative properties have been proposed [39]. Increased levels of TTR after experimental cortical contusion in rats have been demonstrated by proteomic analysis of cortical parenchymal samples and in fact, experimental treatment with thyroxine has been proposed to mitigate some pathophysiologic consequences of TBI [40]. TTR immunopositive cells have been found in corresponding brain territory after middle cerebral artery occlusion and it has been suggested that TTR migrates from CSF to brain parenchyma [41]. Interestingly neuron-derived TTR has recently been proposed to have regulatory effects on astrocytic glycolysis and thereby acting as a metabolism activator [42]. TTR has also been proposed to have regulatory effects of the widespread δ-GABA_A_ receptor and thereby theoretically involving tonic inhibitory pathways in brain [43]. Conflicting results exist however whether brain in situ synthetization exist [24] and although TTR has been shown to increase in the perihemorragic zone after intracerebral haemorrhage monitored by microdialysis techniques [17] very little is known about microdialysis recovery of TTR in other forms of brain injury.

In our results we could demonstrate for the first time in cerebral microdialysate TTR in nine different proteoforms in patients having sustained SAH. The results show a large variation in optical density between different proteoforms indicating that proteoforms may present differently and reflect different physiological aspects in patients. Albeit the generally high values in some proteoforms, there are some vial samples with low values compared to other vials, which may reflect changes in underlying pathology, however sample volume is too small to draw any conclusions from this. Missing data in some observations also make conclusions difficult.

Due to low sample volume we started analysing the material by pooling vials into three time groups based on time from aneurysmal bled. The time frames were set to investigate if there is a temporal evolution in proteoforms over time, which might reflect the pathology of DCI after subarachnoid haemorrhage. The time frames does not, at this point, represent clinically important periods, but may give a clue when designing future studies. In this pooled analysis, the different proteoforms seemed to present a change in optical density related to time from aneurysmal bleed indicating an evolution over time.

Some proteoforms (1101, 4102) showed a strongly increasing pattern over time from aneurysmal bled while others (1102, 2101, 3101) showed a slowly but increasing pattern. A few proteoforms (5001, 5101) showed a slight peak in optical density and thereafter decreasing values. At this point it is not possible to say whether this is a timely evolution of proteoforms after the initial subarachnoid bled or if it represents an increasing synthesis of specific proteoforms as a response or protective means to delayed cerebral ischemia. In this study the pooled groups are too small to draw any conclusions and another weakness in the study is that the pooled groups consist of mixed patient vial samples with no correlation to later development of vasospasm. Further studies in groups with or without clinical signs of vasospasm are needed to further investigate that question.

One patient developed right hemisphere vasospasm shortly after microdialysis sampling was finished. Interestingly in this patient, there was an obvious difference between the two catheter samples from the left hemisphere and the single catheter sample from the right hemisphere with generally much lower optical density values in the right hemisphere in all proteoforms except for 6101. This finding raises questions whether there is a general decrease in many proteoforms, preceding severe vasospasm. However, since there is only one vial sample from the right hemisphere, results may be influenced by artefacts and interpretation should be made with caution.

A secondary aim was to try to confirm the findings of Maurer et al. concerning GAPDH and HSP7C. Due to low sample volume, samples had to be pooled and analyses on HSP7C could not be performed, but analyses on GAPDH showed four different proteoforms. Also these proteoforms seemed to describe a temporal evolution from aneurysmal bled such that 7202 and 7204 decreased clearly over time, while 8203 and 8206 stayed rather unchanged or had an unspecific pattern. Maurer showed that GAPDH increased before development of vasospasm. In our results, although we could not confirm those results from reasons above mentioned, we could find GAPDH in the samples.

## Limitations

This is a small descriptive, hypothesis generating study and due to the nature of the disease and invasiveness of the sampling technique, it is impossible to include healthy controls. The sample size is small which makes statistical validation at this point impossible, but we still find it valuable to publish in order to show that the technique is possible to use for finding new biomarkers. The results in this pilot study should be validated in larger cohorts of patients.

## Conclusions

In this pilot study we found several proteoforms of TTR and their levels differed between the timepoints from aneurysmal bled, showing that proteomic is a useful tool to identify previously unknown possible brain injury biomarkers. Further study to characterize the differences between the proteoforms, their function and timely evolution after aneurysmal bled and their hypothetical correlation to delayed cerebral ischemia after SAH is warranted.

## Supplementary Information

**Additional file 1: Table S1.** Different TTR proteoforms in pooled time groups and single catheters after aneurysmal bled. Unit: Percentage optical density. dm = data missing.

**Additional file 2.**

## Data Availability

The datasets generated and/or analyzed in this study are not  publicly available as the Ethical Review Board has not approved the public availability of these data.
