# Supplementary material for: Altered levels of transthyretin in human cerebral microdialysate after subarachnoid haemorrhage using proteomics; a descriptive pilot study
Source: Proteome Sci. 2023 Jul 7;21:10. doi: 10.1186/s12953-023-00210-z (PMC10326944; doi:10.1186/s12953-023-00210-z)
Supplement: Supplementary file 1 — Additional file 1: Table S1. Different TTR proteoforms in pooled time groups and single catheters after aneurysmal bled. Unit: Percentage optical density. dm = data missing. [file 12953_2023_210_MOESM1_ESM.docx]

**Table S1.** Different TTR proteoforms in pooled time groups and single catheters after aneurysmal bled. Unit: Percentage optical density. dm = data missing
