# Supplementary material for: Altered levels of transthyretin in human cerebral microdialysate after subarachnoid haemorrhage using proteomics; a descriptive pilot study
Source: Proteome Sci. 2023 Jul 7;21:10. doi: 10.1186/s12953-023-00210-z (PMC10326944; doi:10.1186/s12953-023-00210-z)
Supplement: Supplementary file 2 — Additional file 2. [file 12953_2023_210_MOESM2_ESM.pdf]

2019-06444 beslut.pdf

Signers:

Name	*Method*	*Date*
Carin Gunilla Robertsson	BANKID	2020-07-13 14:48 GMT+2

This document package contains:

- Front page (this page)
- The original document(s)
- The electronic signatures. These are not visible in the document, but are electronically integrated.

This file is sealed with a digital signature. The seal is a guarantee for the authenticity of the document.

Document ID:
C79F94A5BE93434F949CC78280ADB291

THE SIGNED DOCUMENT FOLLOWS ON THE NEXT PAGE >

Dnr 2019-06444
Linköping avdelning medicin

BESLUT

Sökande forskningshuvudman
Region Östergötland

Forskare som genomför projektet
Oscar Åneman

Projekttitel
Utvärdering av neuromonitorering på NIVA, US

Aktuell ändring
Ansökan om ändring inkommen 2019-12-18.

Grundansökan godkänd 2014-02-26 av Regionala etikprövningsnämnden i Linköping med diarienummer 2013/471-31.

Etikprövningsmyndigheten beslutar enligt nedan.

BESLUT

Etikprövningsmyndigheten godkänner den forskning som anges i ansökan om ändring.

På Etikprövningsmyndighetens vägnar

Gunilla Robertsson
Ordförande

Beslutet har fattats efter föredragning av vetenskaplig sekreterare Bärbel Jung.

Beslutet sänds till
Ansvarig forskare: Oscar Åneman

This file is sealed with a digital signature.
The seal is a guarantee for the authenticity of the document.
Document ID:
C79F94A5BE93434F949CC78280ADB291
